# Drivers and distribution of the household-level double burden of malnutrition in Bangladesh: analysis of mother–child dyads from a national household survey

**DOI:** 10.1017/S1368980022002075

**Published:** 2022-11

**Authors:** Abdur Razzaque Sarker, Zakir Hossain, Alec Morton

**Affiliations:** 1 Population Studies Division, Bangladesh Institute of Development Studies (BIDS), Room 302, E-17, Sher e Bangla Nagar, Agargaon, Dhaka 1207, Bangladesh; 2 Department of Management Science, University of Strathclyde, Glasgow, UK

**Keywords:** Double burden, Malnutrition, Mother–child dyads, Urban–rural, Bangladesh

## Abstract

**Objective:**

The double burden of malnutrition (DBM) has become an emerging public health issue in many low- and middle-income countries. This study aims to provide important evidence for the prevalence of different types of DBM at the national and subnational levels in Bangladesh.

**Design:**

The study utilised data from the latest Bangladesh Demographic and Health Survey (BDHS) 2017–2018. Multivariable logistic regression was performed to identify the sociodemographic factors associated with DBM.

**Setting:**

Nationally representative cross-sectional survey.

**Participants:**

8697 mothers aged 15 to 49 years with <5 children.

**Results:**

The overall prevalence of the DBM was approximately 21 %, where the prevalence of overweight mother (OWM) & stunted child/wasted child/underweight child (SC/WC/UWC) and underweight mother (UWM) & overweight child (OWC) was 13·35 % and 7·69 %, respectively, with a higher prevalence among urban households (OWM & SC/WC/UWC = 14·22 %; UWM & OWC = 10·58 %) in Bangladesh. High inequality was observed among UWM & OWC dyads, concentration index (CI) = -0·2998, while low level of inequality of DBM were observed for OWM & SC (CI = 0·0153), OWM & WC (CI = 0·1165) and OWM & UWC (CI = 0·0135) dyads. We observed that the age and educational status of the mother, number of children, fathers’ occupation, size and wealth index of the household, and administrative division were significantly associated with all types of DBM.

**Conclusions:**

Health policymakers, concerned authorities and various stakeholders should stress the prevalence of DBM issues and take necessary actions aimed at identifying and addressing the DBM in Bangladesh.

In recent years, the double burden of malnutrition (DBM) has emerged as a global public health issue, particularly for low- and middle-income countries (LMIC)^(1,2)^. According to the WHO, the DBM means the coexistence of undernutrition along with overweight and obesity, within individuals, households and populations, and across the life course^(3)^. Due to rapid urbanisation, economic transition and demographic dividend (i.e. accelerated economic growth offered by the changes in the age structure of a population), many developing countries, including Bangladesh, are experiencing a nutritional transition^(4)^. Children are particularly at risk as poor nutrition in the early years can have lasting consequences throughout life. Yet, various forms of childhood malnutrition, such as stunting, wasting, underweight, overweight and obesity, are very common across various societal groups in many LMIC^(5)^. According to the latest data, globally, approximately 149·2 million, 45·4 million and 38·9 million children aged under 5 years were stunted, wasted and overweight, respectively, while approximately 20 million new-borns were born with low birth weight^(3)^. It was estimated that 45 % of all deaths among children aged unde 5 years were directly related to childhood undernutrition, with most of these deaths occurring in Asia and Africa^(3)^. The number of overweight children (OWC) aged under 5 years increased from 30 million in 2000 to 55·6 million in 2017^(6)^. Further, a number of mothers in the developing world are also suffering from underweight, overweight and obesity-related malnutrition^(7)^. Per the latest estimates, approximately 1·9 billion adults are overweight or obese and 462 million people are suffered underweight globally^(8)^.

Although the prevalence of childhood undernutrition has declined substantially, the prevalence of undernourished children and underweight mothers (UWM) is high in rural areas and among the poorest wealth quintile in Bangladesh^(1,9)^. Further, due to rapid changes in global food systems, increasing urbanisation, decreased physical activity, changes in lifestyle and changes in dietary intake, many developing countries, including Bangladesh, are experiencing overweight-related issues among children and mothers^(7,10)^. As a consequence, the proportion of people with overweight and obesity has increased substantially, particularly among the wealthiest and most educated individuals and people living in urban areas^(1,7)^.

Like other developing countries, Bangladesh is experiencing a coexistence of undernutrition and overweight conditions at the population, individual and household levels, a phenomenon referred to as the DBM, which is an emerging public health problem in Bangladesh^(1)^. According to WHO guidelines, the DBM is characterised by the coexistence of undernutrition (including wasting, stunting and deficiencies in important micronutrients) with overweight, obesity or diet-related non-communicable diseases^(3)^.

The concept of the DBM has emerged in the past three decades and recently received greater attention due to a recent series of papers in *The Lancet*, as it appears to be more permanent and widespread than previously perceived^(11)^. It is well established that both being underweight and overweight have multifaceted consequences for survival, the incidence of chronic diseases, healthy development, and the economic productivity of individuals, societies, and healthcare systems^(12)^. Both overnutrition and undernutrition are equally harmful. Undernutrition often hinders physical and intellectual development, whereas overnutrition is a significant contributor to various non-communicable diseases, including diabetes and hypertension. Both forms of malnutrition cause huge direct and indirect costs to individuals, families and nations; approximately US $3·5 trillion globally^(13)^.

Bangladesh – a lower-middle-income country – has made remarkable progress in improving its population’s health over the past few decades. This may be due to the pluralistic healthcare system in which public providers, private providers and various non-governmental organisations are engaged in healthcare delivery in Bangladesh. As a consequence, the prevalence of childhood stunting (low height for age) was reduced from 51 % in 2004 to 31 % in 2017–2018, while the prevalence of underweight (low weight for age) was reduced from 43 % in 2004 to 22 % in 2017–2018^(14)^. While childhood undernutrition constitutes an enormous burden, the prevalence of childhood overweight conditions (2 % in 2018) is an emerging public health problem in Bangladesh^(14)^. Moreover, in terms of UWM, the prevalence decreased significantly from 30 % in 2007 to 12 % in 2017–2018, while the prevalence of overweight mothers (OWM) has increased alarmingly from 12 % in 2007 to 32 % in 2017–2018^(14)^. Recently, an increasing trend of overweight or obese has been observed among urban and wealthier individuals in Bangladesh^(15)^. It has been noted that UWM were found to coexist with OWC, and OWM were found to coexist with stunting, wasting, and underweight conditions in children within the same households in Bangladesh. The WHO policy brief on DBM indicated that most current policies tend to address either undernutrition or overweight and obesity, but not both; therefore, actions that address both conditions should be prioritised globally^(3)^. Such double-duty actions include interventions, programmes and policies that have the potential to simultaneously lessen the risk or burden of both undernutrition and overnutrition. This is an urgent issue, as the coexistence of various forms of malnutrition among mothers and children has continued to rise not only in Bangladesh but globally. Notably, malnourished women are susceptible to experiencing complications related to pregnancy and childbirth^(16)^.

A number of studies have identified the DBM in various settings globally^(1,5)^. A recent multi-country study that included Bangladesh estimated the DBM using three separate combinations of overweight or obese mothers with undernourished children (i.e. underweight children (UWC), stunted children (SC) and wasted children (WC))^(17)^. It was also observed that the prevalence of UWM remained high in rural areas, while the prevalence of OWM increased rapidly in both rural and urban areas, creating a DBM among mothers in Bangladesh^(1)^. Another study predicted that by the year 2030, the prevalence of UWM would be highest among the poorest segment of society, and the prevalence of overweight and obesity would be highest among the richest segment in Bangladesh^(5)^. A multi-country study conducted in Bangladesh, Nepal, Pakistan, and Myanmar referred to household-level DBM as the coexistence of OWM and SC in the same household only but did not focus on the other types of DBM^(18)^. To the best of our knowledge, analysis of the UWM along with the overweight and obesity status of the children and the OWM along with the stunting, wasting and underweight status of the children in the same household to explore the status of DBM using nationally representative data in Bangladesh has not yet been performed. This study aims to provide important information about the prevalence of various forms of DBM at the national level and by urbanity in Bangladesh. The specific objectives of the study are to measure the prevalence, inequality and factors associated with the overall DBM at the household level in Bangladesh.

## Materials and methods

### Study population and data source

The study utilised data from the most recent Bangladesh Demographic and Health Survey (BDHS) 2017–2018. The survey was carried out from October 2017 to March 2018 under the authority of the National Institute of Population Research and Training, Medical Education and Family Welfare Division, and Ministry of Health and Family Welfare^(14)^. The BDHS is a vital source of records of data used in this study, including women’s BMI and records of stunting, wasting, underweight, and overweight conditions of children under 5 years of age. Women aged 15 to 49 years with at least one of their children living in the same household were the population of this study.

### Survey design and sampling procedures

The BDHS 2017–2018 was a cross-sectional survey that used a two-stage stratified random sampling design to cover the entire population by taking a nationally representative sample. This survey used a list of enumeration areas and a standard sampling frame provided by the Bangladesh Bureau of Statistics^(14)^. A total of 8772 individual mothers aged 15 to 49 years were enrolled in this study. However, seventy-five mothers were excluded, because no children lived in their households. Thus, a total of 8697 samples were analysed, with 2379 and 6319 mothers from urban and rural areas, respectively.

### Outcome variables

The outcome variable was the prevalence of the DBM at the household level, disaggregated by urban and rural households. The DBM was defined as the coexistence of mothers’ underweight condition and children’s overweight condition or the coexistence of mothers’ overweight condition and children’s stunting, wasting, or underweight condition in the same household^(3)^. Mothers’ nutritional burden was defined as the existence of underweight or overweight conditions in the mother, while children’s nutritional burden was defined as stunting, wasting, underweight or overweight conditions. The 2017–2018 BDHS used the WHO’s guidelines to determine the cut-off values for mothers’ BMI (i.e. underweight and overweight) and the stunting, wasting, underweight and overweight status of the children^(14)^.

### Explanatory variables

A number of explanatory variables were included in this study based on relevance and logical correlation with the DBM among women and children globally^(1,2,5)^. The series of explanatory variables were as follows: age and educational and working status of the mother, mother’s age at first birth, number of children, father’s education and occupation, sex of children, child’s birth order, household size, toilet facilities, respondent’s exposure to mass media, wealth index, and administrative division of the households. The households of the study participants were categorised based on whether they were residing in urban or rural areas. Respondents’ age was categorised into three groups: 15–19 years, 20–29 years and 30–49 years. Maternal and paternal educational status were reported by the study participants and categorised as ‘no formal education’, ‘primary’, ‘secondary’ and ‘higher’. Mother’s age at first birth was categorised into three groups (less than 18, 18–24 and 25 or above), and working status of the mothers was categorised as ‘yes’ and ‘no’. Likewise, the respondent’s number of children was categorised into three groups (one child, two children, and three or more children). A composite score named the ‘wealth index’ was calculated using principal component analysis based on the household’s ownership of selected assets, availability of electricity supply, television, bicycle, materials used for housing construction, types of water access and sanitation facilities, use of health and other services, and health outcomes. Ultimately, the wealth index was used to categorise households into the ‘poorest’, ‘poorer’, ‘middle’, ‘richer’ and ‘richest’ quintiles^(14)^.

### Measurement of inequality

Measurement of inequality was performed using the concentration curves and concentration indices. The concentration curve provides the distribution of DBM among the socio-economic groups. The cumulative proportion of DBM in the vertical axis is depicted against the cumulative proportion of the samples regarding socio-economic status. If the DBM is more concentrated among poor people, the concentration curve will lie above the equity line and vice versa. If the concentration curve equals the 45-degree straight line exactly, this means that there is perfect equity in DBM with respect to the wealth index. The wealth index was calculated through principal component analysis using BDHS survey data. The concentration index (CI) shows the information contained in each concentration curve and is twice the area between the concentration curves and the equity line^(19)^. The value of the CI lies between –1 and +1 (i.e. –1 ≤ CI ≤ + 1), where –1 refers to the case where DBM is entirely concentrated among the poorest quintile, and +1 refers to the case where malnutrition is entirely concentrated among the wealthiest quintile. Further, a value of 0 (zero) signifies perfect equality, i.e. there is no socio-economic inequity for the DBM.

### Statistical analysis

Descriptive analysis, such as frequency distribution and cross-tabulation, was applied for measuring the prevalence of DBM according to background variables. The inequality of DBM was measured by generating the Lorenz curve using Microsoft Office Excel version 16.0. Both adjusted and unadjusted logistic regression models were used to examine the significant risk factors. The dependent variable was expressed as binary, and it was represented as ‘1’ for the coexistence of mothers’ underweight condition and children’s overweight condition or the coexistence of mothers’ overweight condition and children’s stunting, wasting or underweight condition in the same household, while ‘0’ was represented for the non-coexistence of mothers’ underweight condition and children’s overweight condition or the coexistence of mothers’ overweight condition and children’s stunting, wasting or underweight condition in the same household. In the multivariable logistic regression models, results were presented as adjusted OR (AOR) with 95 % CIs. Results were considered to be statistically significant at the 5 % *α* level (*P* < 0·05). Since the BDHS survey used a two-stage stratified cluster sampling technique, the recommended sample weights provided by the BDHS were used for the analysis. All statistical analyses were carried out using the statistical package Stata/SE 14 software (Stata Corp.).

## Results

### Sociodemographic characteristics of the study participants

The background characteristics of the study participants are described in Table 1. A total of 8697 women with at least one child aged up to 60 months were included in this analysis, with most of the participants living in rural areas (72·65 %). More than half of the respondents were young adults aged 20 to 29 years (62·41 %) and had completed secondary and higher education (63·8 %). Approximately 46 % and 37 % of rural and urban mothers, respectively, had their first pregnancy before 18 years of age, while 65 % of mothers had at least two children. Approximately 59 % of mothers had a BMI within the normal range, while 27 % were overweight or obese and 14 % were underweight. Approximately 52 % of the children were male. Approximately 31 % of the children were stunted, followed by underweight (22 %), wasted (8 %), obese (8 %) and overweight (2 %). Approximately one-third (31 %) of the study households were large in size (six or more family members), while only 13 % of the study households were small in size (<4 family members). Most of the participants (60 %) were using hygienic toilet facilities, and 42 % of the participants had access to mass media. According to the wealth index, 26 % of the rural population enrolled in the study were from the poorest quintile, and 45 % of the urban population enrolled in the study were from the richest quintile. The highest number of participants (26 %) belonged to the Dhaka division (largest administrative unit), followed by the Chittagong division (21 %), while the lowest number of participants (6 %) belonged to the Barisal division.

**Table 1:** Sociodemographic characteristics of the study participant (*n* 8697)

Variables	Urban (*n* 2379)		Rural (*n* 6319)		Overall (*n* 8697)	
	Frequency	%	Frequency	%	Frequency	%
Double burden (all types)						
Yes	590	24·79	1240	19·62	1829	21·03
Mothers age						
15–19 years	275	11·58	833	13·19	1109	12·75
20–29 years	1463	61·51	3965	62·75	5428	62·41
30–49 years	640	26·92	1520	24·06	2161	24·84
Mothers’ educational status						
No formal education	178	07·48	466	07·37	644	07·40
Primary	610	25·66	1894	29·98	2505	28·80
Secondary	1056	44·38	3156	49·94	4212	48·42
Higher	535	22·48	803	12·70	1337	15·38
Mothers age at first birth						
Less than 18 years	871	36·61	2915	46·13	3785	43·52
18–24 years	1306	54·91	3180	50·33	4486	51·58
25 years or more	202	08·48	224	03·55	426	04·90
Number of children						
One child	927	38·96	2101	33·25	3028	34·82
Two children	888	37·33	2300	36·40	3188	36·66
Three or more child	564	23·71	1917	30·34	2481	28·53
Mothers BMI						
Underweight	258	10·84	926	14·65	1183	13·61
Normal weight	1189	49·96	3972	62·87	5161	59·34
Overweight	656	27·57	1101	17·43	1757	20·20
Obese	277	11·63	319	05·06	596	06·85
Mothers working status						
Yes	772	32·44	2774	43·91	3546	40·77
Fathers’ education						
No formal education	306	12·84	1155	18·28	1461	16·80
Primary	699	29·40	2238	35·43	2938	33·78
Secondary	763	32·08	2034	32·18	2797	32·16
Higher	611	25·67	891	14·11	1502	17·27
Fathers’ occupation						
Day labour	1439	60·48	4650	73·59	6089	70·01
Business	643	27·02	1166	18·46	1809	20·80
Service	229	09·61	254	04·01	482	05·55
Unemployed	16	00·68	50	00·79	66	00·76
Others	52	02·20	199	03·15	251	02·89
Sex of children						
Male	1207	50·74	3333	52·75	4540	52·20
Female	1172	49·26	2985	47·25	4157	47·80
Child stunting (*n* 7818)						
Yes	524	25·32	1877	32·66	2402	30·72
Child wasting (*n* 7804)						
Yes	186	09·02	473	08·24	659	08·44
Child underweight (*n* 8041)						
Yes	407	19·10	1345	22·77	1753	21·80
Child overweight (*n* 7781)						
Yes	45	02·20	72	01·25	117	01·50
Child obesity						
Yes	253	10·65	419	06·63	672	07·73
Birth order						
First	996	41·86	2340	37·04	3336	38·36
Second	799	33·58	1989	31·48	2788	32·05
Third or more	584	24·56	1989	31·48	2573	29·59
Household size						
Small (<4)	421	17·72	722	11·42	1143	13·14
Medium (4–6)	1347	56·62	3508	55·51	4855	55·82
Large (6 and more)	610	25·66	2089	33·06	2700	31·04
Division						
Dhaka	1109	46·63	1128	17·85	2237	25·73
Chittagong	466	19·60	1343	21·25	1809	20·80
Rajshahi	182	07·65	828	13·10	1010	11·61
Rangpur	133	05·61	786	12·44	920	10·57
Khulna	184	07·74	612	09·69	796	09·16
Mymensingh	122	05·12	604	09·56	726	08·34
Sylhet	104	04·37	612	09·69	716	08·23
Barisal	78	03·28	406	06·42	484	05·56
Toilet facility						
Hygienic	1871	78·64	3389	53·64	5260	60·48
Unhygienic	508	21·36	2929	46·36	3437	39·52
Mass media exposure						
Yes	1501	63·08	2184	34·56	3684	42·36
Wealth index						
Poorest	206	08·64	1669	26·41	1874	21·55
Poorer	149	06·24	1617	25·59	1766	20·30
Middle	279	11·72	1353	21·42	1632	18·77
Richer	672	28·24	1073	16·97	1744	20·05
Richest	1074	45·16	607	09·60	1681	19·33
Total	2379	27·35	6319	72·65	8697	100

### Prevalence of double burden of malnutrition across background characteristics

The prevalence of DBM across background characteristics is described in Table 2. The overall prevalence of DBM characterised by OWM & SC/WC/UWC and UWM & OWC was 13·35 % and 7·69 %, respectively. Although a similar pattern was found for both OWM’s dyad with undernourished child (urban: 14·22 % and rural: 13·02 %) and UWM’s dyad with OWC (urban: 10·58 % and rural: 6·60 %), the differences between urban and rural were much higher among the UWM & OWC dyad. The prevalence of DBM increased gradually as mothers’ age increased, and the highest DBM was found among the mothers aged 30–49 years (OWM & SC/WC/UWC: 15·89 % and UWM & OWC: 9·14 %). The highest prevalence of DBM (OWM & SC/WC/UWC: 15·58 % and UWM & OWC: 11·49 %) was noticed among the mothers who had no formal education, while the urban–rural difference was found higher among OWM & SC/WC/UWC dyads (urban: 19·86 % and rural: 13·94 %) and the lowest prevalence was found for the highest educated mothers. We found that mothers in urban areas who had their first child after age 24 were more prone to both OWM & SC/WC/UWC (15·66 %) and UWM & OWC (13·47 %) dyads. Such pattern was not observed among rural mothers.

**Table 2:** Prevalence of double burden of malnutrition (OWM & SC/WC/UWC, UWM & OWC) by sociodemographic characteristics

Variables	OWM & SC/WC/UWC						UWM & OWC					
	Urban		Rural		Overall		Urban		Rural		Overall	
	*n*	%	*n*	%	*n*	%	*n*	%	*n*	%	*n*	%
Mothers age												
15–19 years	31	11·15	81	09·67	111	10·04	22	08·01	48	05·72	70	06·29
20–29 years	199	13·62	507	12·78	706	13·01	156	10·67	245	06·19	401	07·40
30–49 years	108	16·91	235	15·46	343	15·89	73	11·47	124	08·15	197	09·14
*P*-value	0·025		0·001		0·000		0·394		0·023		0·016	
Mothers’ educational status												
No formal education	35	19·86	65	13·94	100	15·58	22	12·38	52	11·15	74	11·49
Primary	98	16·09	239	12·61	337	13·46	67	11·00	142	07·49	209	08·34
Secondary	146	13·84	429	13·59	575	13·65	109	10·30	175	05·55	284	06·75
Higher	59	10·94	90	11·20	148	11·09	54	10·03	48	05·97	102	07·60
*P*-value	0·033		0·269		0·029		0·401		0·001		0·001	
Mothers age at first birth												
Less than 18 years	131	15·05	360	12·35	491	12·98	94	10·8	226	07·74	320	08·44
18–24 years	175	13·43	437	13·75	613	13·66	130	09·98	176	05·52	306	06·82
25 years or more	32	15·66	25	11·24	57	13·34	27	13·47	16	07·10	43	10·12
*P*-value	0·172		0·053		0·681		0·329		0·007		0·005	
Number of children												
One child	110	11·88	248	11·78	358	11·82	122	13·13	165	07·84	287	09·46
Two children	131	14·77	288	12·54	420	13·16	74	08·31	123	05·35	197	06·17
Three or more child	97	17·17	287	14·95	383	15·45	56	09·94	129	06·74	185	07·47
*P*-value	0·008		0·015		0·000		0·002		0·005		0·000	
Mothers working status												
No	229	14·27	489	13·80	718	13·94	140	08·71	226	06·37	366	07·10
Yes	109	14·11	333	12·02	442	12·47	112	14·45	191	06·90	303	08·54
*P*-value	0·842		0·011		0·047		0·000		0·660		0·035	
Fathers’ education												
No formal education	46	15·12	150	13·00	196	13·44	33	10·64	108	09·38	141	09·65
Primary	105	14·97	274	12·26	379	12·90	71	10·12	135	06·04	206	07·01
Secondary	115	15·01	288	14·14	402	14·38	70	09·12	120	05·89	189	06·77
Higher	73	11·90	110	12·39	183	12·19	79	12·88	54	06·02	132	08·81
*P*-value	0·073		0·635		0·338		0·051		0·000		0·000	
Fathers’ occupation												
Day labour	210	14·56	630	13·55	840	13·79	147	10·21	293	06·29	439	07·22
Business	91	14·16	134	11·47	225	12·42	67	10·37	74	06·35	141	07·78
Service	31	13·46	25	09·75	56	11·51	26	11·44	22	08·57	48	09·93
Others	02	09·88	06	12·72	08	12·03	02	09·66	05	10·30	07	10·14
Unemployed	05	09·97	28	13·82	33	13·02	10	19·78	24	11·83	34	13·49
*P*-value	0·776		0·301		0·613		0·030		0·004		0·000	
Sex of children												
Male	183	15·15	427	12·81	610	13·43	126	10·48	224	06·73	351	07·73
Female	155	13·25	396	13·25	551	13·25	125	10·67	193	06·45	318	07·64
*P*-value	0·313		0·561		0·902		0·700		0·979		0·738	
Birth order												
First	118	11·81	283	12·10	401	12·01	118	11·81	154	06·57	271	08·13
Second	121	15·19	258	12·96	379	13·60	70	08·73	116	05·81	185	06·65
Third or more	99	16·98	282	14·16	381	14·80	64	11·00	148	07·43	212	08·24
*P*-value	0·005		0·134		0·004		0·117		0·223		0·068	
Household size												
Small (<4)	61	14·47	101	14·01	162	14·18	85	20·06	75	10·39	159	13·95
Medium (4–6)	189	14·06	455	12·97	644	13·28	116	08·64	208	05·93	324	06·68
Large (6 and more)	88	14·39	266	12·75	354	13·12	51	08·30	134	06·41	185	06·84
*P*-value	0·866		0·841		0·898		0·000		0·000		0·000	
Division												
Dhaka	179	16·10	131	11·63	310	13·85	156	14·10	77	06·81	233	10·42
Chittagong	61	13·14	194	14·46	255	14·12	43	09·21	99	07·35	142	07·83
Rajshahi	20	10·90	132	15·91	152	15·01	14	07·77	46	05·60	60	05·99
Rangpur	21	15·56	81	10·28	102	11·05	08	05·97	45	05·78	53	05·81
Khulna	18	09·54	70	11·37	87	10·95	13	06·88	34	05·60	47	05·90
Mymensingh	19	15·90	61	10·16	81	11·12	05	04·45	39	06·51	45	06·17
Sylhet	10	09·62	96	15·77	106	14·87	08	07·55	46	07·50	54	07·51
Barisal	11	13·74	57	14·10	68	14·04	04	05·40	30	07·44	34	07·11
*P*-value	0·018		0·001		0·101		0·000		0·507		0·000	
Toilet facility												
Hygienic toilet facility	274	14·63	465	13·71	738	14·04	209	11·17	200	05·90	409	07·77
Unhygienic toilet facility	65	12·69	358	12·22	422	12·29	43	08·39	217	07·41	260	07·55
*P*-value	0·262		0·041		0·019		0·107		0·041		0·807	
Mass media exposure												
Yes	219	14·58	306	13·99	524	14·23	159	10·60	129	05·92	288	07·83
No	119	13·59	517	12·50	636	12·69	92	10·53	288	06·96	380	07·59
*P*-value	0·318		0·038		0·024		0·942		0·062		0·874	
Wealth index												
Poorest	26	12·58	207	12·38	232	12·40	16	07·87	126	07·53	142	07·57
Poorer	25	17·12	176	10·91	202	11·43	9	05·96	115	07·10	124	07·00
Middle	33	11·66	177	13·07	209	12·83	20	07·11	72	05·28	91	05·59
Richer	109	16·28	172	16·06	282	16·14	75	11·10	62	05·77	136	07·82
Richest	145	13·50	90	14·90	235	14·00	132	12·31	43	07·10	175	10·43
*P*-value	0·319		0·002		0·009		0·012		0·213		0·001	
Overall	338	14·22	823	13·02	1161	13·35	252	10·58	417	06·60	669	07·69
*P*-value	0·730						0·000					

OWM, overweight mother; SC, stunted child; WC, wasted child; UWC, underweight child; UWM, underweight mother; OWC, overweight child.

DBM in terms of OWM & SC/WC/UWC dyads (15·45 %) was most prevalent among the mothers who had three or more children at the time of the survey. However, a different scenario was found for the UWM & OWC dyads and mothers with a single child (10 %) were more prone to DBM, while the scenario was more common in urban areas (13·13 %) than rural areas (7·84 %). The results indicated that the DBM was more prevalent among the children whose fathers had no formal education than among those whose fathers had a higher educational level. Male children had a slightly higher prevalence of the DBM than female children, while urban male children had suffered more (OWM & SC/WC/UWC dyads, 15·15 %, and UWM & OWC dyads, 10·48 %) than rural male children. Children whose birth order was third or more were more prone to DBM in both urban and rural areas for all dyads. The prevalence of DBM was found to be highest among the small (<4 family members) households (OWM & SC/WC/UWC: 14·18 % and UWM & OWC: 13·95 %) compared to both the medium (4–6 family members) and large (six or more family members) households. However, the differences between urban and rural were much higher among UWM & OWC dyads, particularly for small households. In terms of division, Dhaka was found to be the most prevalent for UWM & OWC dyads (10·42 %), while the OWM & SC/WC/UWC dyads were more common in Sylhet division (15 %). The prevalence of DBM characteristics by UWM & OWC dyads was highest (10·43 %) among the richest households, while the prevalence of OWM & SC/WC/UWC dyads was high among richer households (16·14 %) followed by the richest households (14 %).

### Prevalence of various forms of double burden of malnutrition among residents of rural and urban areas

**Fig. 1:**
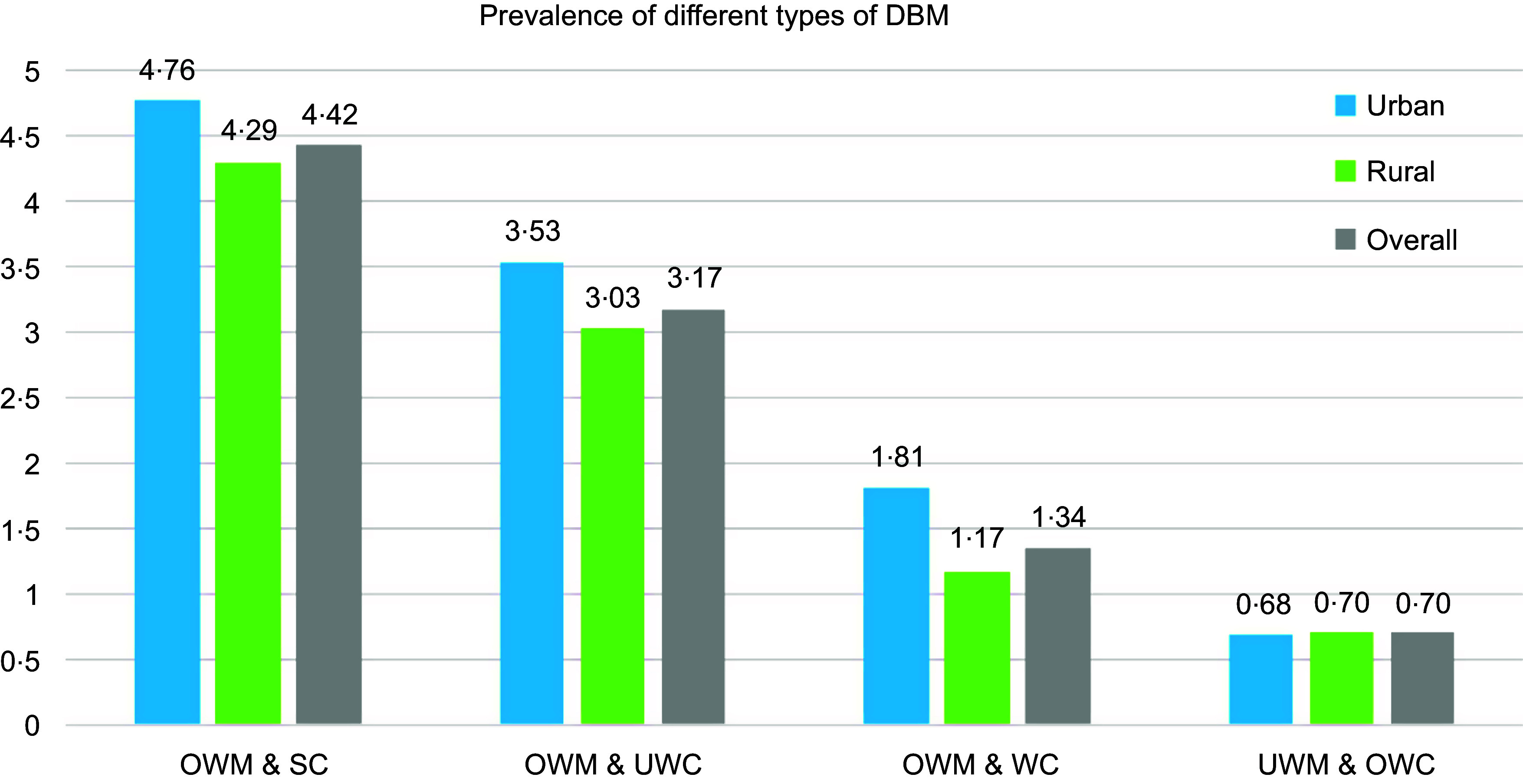
Prevalence of various forms of DBM among residents of rural and urban areas. DBM, double burden of malnutrition; OWM, overweight mother; SC, stunted child; WC, wasted child; UWC, underweight child; UWM, underweight mother; OWC, overweight child

The prevalence of various forms of DBM is shown in Fig. 1. The DBM paired households were categorised as: OWM & SC; OWM & UWC; OWM & WC; and UWM & OWC. The overall prevalence of DBM was highest for OWM & SC (4·42 %), followed by OWM & UWC (3·17 %). In urban areas, the prevalence of OWM & SC was higher (4·76 %) than the overall prevalence of this pair and was the most common pair, followed by OWM & UWC (3·53 %). In the rural areas, OWM & SC (4·29 %) and OWM & UWC (3·03 %) were more prevalent DBM pairs.

### Inequality in the prevalence of different types of double burden of malnutrition

**Fig. 2:**
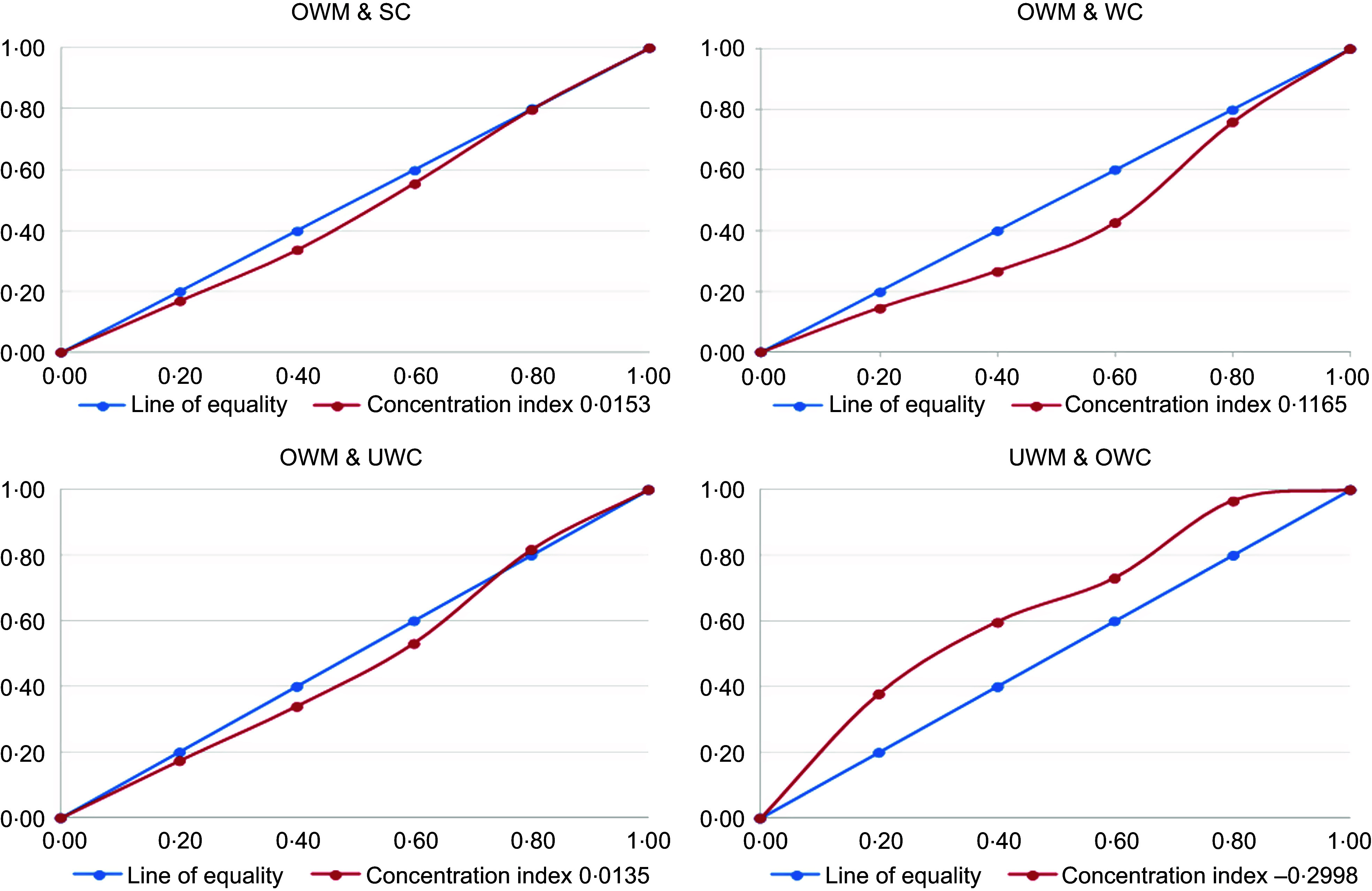
Inequality in the prevalence of different types of double burden of malnutrition. OWM, overweight mother; SC, stunted child; WC, wasted child; UWC, underweight child; UWM, underweight mother; OWC, overweight child

Figure 2 shows the inequality of the prevalence of various forms of DBM using concentration curves. High inequality was observed among the UWM & OWC (CI -0·3) dyad, which indicated that poor households were more vulnerable to this type of DBM. A low level of inequality of DBM were observed for OWM & SC (CI 0·015), OWM & WC (CI 0·116) and OWM & UWC (CI 0·013) dyads.

### Factors associated with the double burden of malnutrition

Table 3 shows the various risk factors associated with the DBM (OWM & SC/WC/UWC and UWM & OWC) across background characteristics. We observed that the age and educational status of the mother, number of children, fathers’ occupation, size of the household, administrative division and wealth index of the household were significantly associated with the OWM & SC/WC/UWC dyads, but no significant associations were found for fathers’ education, place of residence and birth order of the child with the same dyads in the adjusted model. We observed a positive relationship between the age of the mother and DBM for such dyads. The risk of DBM was 1·36 (95 % CI (1·08, 1·71); *P* = 0·01) and 1·73 (95 % CI (1·30, 2·30); *P* = 0·001) times higher among individual mothers aged 20–29 years and 30–49 years, respectively, than the reference age group (mothers aged 15–19 years). Uneducated mothers (AOR 1·71; 95 % CI (1·21, 2·40); *P* = 0·001), educated mothers who completed primary education (AOR 1·45; 95 % CI (1·11, 1·90); *P* = 0·01) and secondary education (AOR 1·39; 95 % CI (1·10, 1·74); *P* = 0·01) were more likely to exhibit the DBM compared to higher-educated mothers, and this difference was statistically significant for OWM & SC/WC/UWC dyads. However, mothers having two children (AOR 0·76; 95 % CI (0·57, 1·00); *P* = 0·03) and fathers doing business (AOR 0·83; 95 % CI (0·71, 0·98); *P* = 0·03) were less likely to DBM for such dyads. A higher risk of DBM was observed among the small households (AOR 1·30; 95 % CI (1·05, 1·62); *P* = 0·01) compared to the larger households. According to the administrative divisions, the DBM was less prevalent in the Rangpur division (AOR 0·75; 95 % CI (0·57, 0·98); *P* = 0·03), Khulna division (AOR 0·68; 95 % CI (0·51, 0·91); *P* = 0·01) and Mymensingh (AOR 0·72; 95 % CI (0·54, 0·96); *P* = 0·03) than the reference division (Rajshahi) for OWM & SC/WC/UWC dyads. In addition, such DBM was more common among richer (AOR 1·46; 95 % CI (1·18, 1·81); *P* = 0·001) and richest (AOR 1·38; 95 % CI (1·06, 1·78); *P* = 0·01) households compared to the poorest households.

**Table 3:** Factors associated with household-level double burden of malnutrition (OWM & SC/WC/UWC, UWM & OWC) among mother–child dyads in Bangladesh

Variables	OWM & SC/WC/UWC (*n* 8038)		UWM & OWC (*n* 7537)	
	AOR	95 % CI	AOR	95 % CI
Mothers age				
15–19 years (ref.)				
20–29 years	1·36**	1·08, 1·71	1·50**	1·13, 2·01
30–49 years	1·73***	1·30, 2·30	1·97***	1·38, 2·83
Mothers’ educational status				
No formal education	1·71***	1·21, 2·40	2·29***	1·51, 3·48
Primary	1·45**	1·11, 1·90	1·76***	1·25, 2·48
Secondary	1·39**	1·10, 1·74	1·35*	1·01, 1·81
Higher (ref.)				
Number of children				
One child	0·71	0·49, 1·04	3·27***	2·08, 5·14
Two children	0·76*	0·57, 1·00	1·42*	1·00, 2·01
Three or more child (ref.)				
Fathers’ education				
No formal education	0·95	0·71, 1·27	0·91	0·64, 1·31
Primary	0·94	0·74, 1·21	0·78	0·57, 1·07
Secondary	1·07	0·85, 1·35	0·79	0·59, 1·06
Higher (ref.)				
Fathers’ occupation				
Day labour (ref.)				
Business	0·83*	0·71, 0·98	1·06	0·86, 1·31
Service	0·87	0·62, 1·23	1·22	0·83, 1·80
Unemployed	0·79	0·37, 1·69	1·27	0·55, 2·90
Others	1·01	0·69, 1·50	1·79**	1·19, 2·70
Place of residence				
Urban	1·03	0·87, 1·21	1·32**	1·07, 1·62
Rural (ref.)				
Birth order				
First (ref.)				
Second	0·97	0·75, 1·26	1·55**	1·13, 2·14
Third or more	0·77	0·53, 1·12	2·36***	1·51, 3·69
Household size				
Small (<4)	1·30**	1·05, 1·62	1·91***	1·48, 2·45
Medium (4–6)	1·01	0·88, 1·17	0·96	0·79, 1·17
Large (6 and more) (ref.)				
Division				
Dhaka	0·86	0·69, 1·07	1·48**	1·09, 2·02
Chittagong	0·90	0·72, 1·13	1·39*	1·01, 1·93
Rajshahi (ref.)				
Rangpur	0·75*	0·57, 0·98	0·94	0·64, 1·39
Khulna	0·68**	0·51, 0·91	0·95	0·64, 1·42
Mymensingh	0·72*	0·54, 0·96	0·98	0·65, 1·47
Sylhet	0·95	0·72, 1·26	1·21	0·81, 1·80
Barisal	1·00	0·73, 1·37	1·25	0·80, 1·96
Wealth index				
Poorest (ref.)				
Poorer	0·94	0·76, 1·15	1·00	0·77, 1·30
Middle	1·07	0·87, 1·33	0·75	0·56, 1·01
Richer	1·46***	1·18, 1·81	0·99	0·74, 1·31
Richest	1·38**	1·06, 1·78	1·24	0·89, 1·72
Constant	0·12***	0·07, 0·21	0·01***	0·01, 0·03
*n*	8038		7537	
LR *χ*^2(30)^	99·48		217·97	
Prob > *χ*^2^	0·000		0·000	
Pseudo *R*^2^	0·015		0·0483	
Log likelihood	−3267·52		−2148·57	
Mean VIF	3·10		3·04	

OWM, overweight mother; SC, stunted child; WC, wasted child; UWC, underweight child; UWM, underweight mother; OWC, overweight child; AOR, adjusted odds ratio (control factors: mother age at first birth, working status of mothers, sex of children, type of toilet facility and exposure of mass media).

* *P* < 0·05.

** *P* < 0·01.

*** *P* < 0·001.

The age and educational status of the mother, the number of children, the fathers’ occupation, place of residence, birth order of the children, the size of the household and administrative division were significantly associated with the UWM & OWC dyads in the adjusted model. The prevalence of DBM characteristics by UWM & OWC dyads was highest among the mothers aged 30–49 years old (AOR 1·97; 95 % CI (1·38, 2·83); *P* = 0·001) and the mothers aged 20–29 years old (AOR 1·50; 95 % CI (1·13, 2·01); *P* = 0·01), respectively. Uneducated mothers (AOR 2·29; 95 % CI (1·51, 3·48); *P* = 0·001), primarily educated mothers (AOR 1·76; 95 % CI (1·25, 2·48); *P* = 0·001) and secondary educated mothers (AOR 1·35; 95 % CI (1·01, 1·81); *P* = 0·03) were significantly more likely to manifest the UWM & OWC dyads compared to higher-educated mothers. Mothers having one child were 3·27 times (95 % CI (2·08, 5·14); *P* = 0·001) and two children were 1·42 times (95 % CI (1·00, 2·01); *P* = 0·03) more likely to encounter DBM for such dyads compared to the reference group (three or more child) where the urban households were more prone to DBM characterised by maternal undernutrition and child overnutrition (AOR 1·32; 95 % CI (1·07, 1·62); *P* = 0·01) than the rural areas for such dyads. According to birth order, second and third or more child were 1·55 and 2·36 times significantly more likely to exhibit DBM, respectively, for UWM & OWC dyads. We noticed a positive relationship between the small households (AOR 1·91; 95 % CI (1·48, 2·45); *P* = 0·001) and DBM compared to the larger households for similar dyads. Dhaka (AOR 1·48; 95 % CI (1·09, 2·02); *P* = 0·01) and Chittagong (AOR 1·39; 95 % CI (1·01, 1·93); *P* = 0·03) divisions were more likely to exhibit DBM characterised by UWM & OWC dyads.

## Discussion

Although Bangladesh has made substantial progress in reducing childhood undernutrition in the past decade, the rapid rise in overweight condition is a major challenge. Further, the prevalence of malnutrition among women of reproductive age is a major concern in Bangladesh^(17)^. To our knowledge, this is the first study examining the prevalence of DBM that considers all forms of pairwise coexistence of malnutrition among mothers and children at the household level using nationwide representative data. Our findings provide a new perspective to researchers, policymakers and public health agencies, who can take initiatives to reduce this emerging public health burden in Bangladesh.

This study observed that the overall prevalence of the DBM at the household level was approximately 21 %, where the prevalence of OWM & SC/WC/UWC and UWM & OWC was 13·35 % and 7·69 %, respectively, with a significantly higher prevalence among urban households in Bangladesh. Bangladesh is experiencing rapid urban population growth; nonetheless, urban health is often neglected^(20)^. Further, the large-scale unplanned rural–urban migration resulted in overloaded public services, scarcity of housing, inappropriate diets, unreachable healthcare facilities, and an adverse impact on health and the environment in many urban settings in Bangladesh^(21)^. In addition, various restaurants, supermarkets and food parks are gaining popularity in urban Bangladesh, serving as places for recreational family activities^(22)^. This changes the everyday food intake of urban residents, and junk food or ultra-processed food consumption was notably high among these residents due to various enabling factors, such as addictive taste, changing lifestyles, propagandist advertising and instant availability, while it resulted in obesity in children but also undernutrition in mothers because of the lack of essential nutrients for normal growth^(23)^. According to the latest urban health survey, only a negligible improvement in childhood nutritional status was observed over the last 7 years, with maternal health indicators being particularly unsatisfactory among slum dwellers, who comprise one-third of the urban population in Bangladesh^(24)^. There are approximately fourteen thousand urban slums in Bangladesh, and these areas exhibit many factors that negatively affect the health and nutrition of both mothers and their children^(25)^. Other studies found a positive association between urbanisation and BMI in various settings^(26,27)^.

A recent systematic review indicated an increasing trend in overweight and obesity among children, adolescents, and adults over time, with a higher prevalence in urban areas of Bangladesh^(15)^. A recent study observed that the prevalence of overweight was significantly higher in women (79 % *v*. 53 %) than in men in urban Bangladesh^(28)^. In contrast, rural mothers are more prone to underweight than urban mothers in Bangladesh, as has been observed in other resource-poor countries^(29,30)^. A recent multi-country study reported that women living in rural communities had a greater risk of having UWC than urban mothers^(31)^. Further, the prevalence of childhood undernutrition is more common in rural than in urban communities in many settings^(9,29)^. Previous studies observed that the prevalence of childhood malnutrition was higher among Bangladeshi rural children, a phenomenon that has been frequently observed in other developing countries^(9,17)^

This study assessed four different forms of DBM at the household level: UWM & OWC, OWM & SC, OWM & WC, and OWM & UWC. Between 2004 and 2014, there was a 15 % increase in the prevalence of overweight status and a similar decrease in the underweight status of women of reproductive age. The reduction in underweight status was of similar magnitude in both urban and rural areas, whereas there was a greater relative change in overweight status in the rural areas, which is congruent with recent review findings^(32)^. The underweight prevalence in rural areas remained relatively high, as did the overweight prevalence among urban residents. These findings, indicating a shift of nutritional burdens, are an extension of previous findings, demonstrating consistency with the literature from Bangladesh^(1,33)^. Similar to what had previously been observed in many LMIC, this study found that the OWM & SC pair was the most prevalent DBM at the household level^(17,34)^. Compared with the neighbouring countries, the prevalence of OWM & SC we observed is lower than that previously reported in India (8 %) and Pakistan (24 %)^(17)^. Likewise, a higher prevalence of OWM & SC was also observed in many African and Latin American countries, including Egypt (12·5 %), Ghana (12·5 %), Nicaragua (12·5 %), Bolivia (15 %), Peru (16 %) and Guatemala (23 %)^(34)^. Although our study did not attempt to identify the underlying reason for this difference, increases in the prevalence of overweight women in South and Southeast Asia in recent decades appear to be an important factor^(35)^. Regarding the OWM & UWC pair, our results were in accordance with reports from 18 LMIC in South Asia, Africa and Latin America, where the prevalence ranged from 0·3 % to 5·3 %^(35)^. This study found that the prevalence of OWM & WC at the household level in Bangladesh was lower than that observed in other settings like Nepal (5 %), Myanmar (6 %), India (7 %), Maldives (12 %) and Pakistan (14 %)^(34)^. This is likely because the prevalence of wasting (8 %) is much lower than that of stunting (31 %) and underweight (22 %) among Bangladeshi children^(14)^.

This study highlights the socio-economic inequality of the DBM, particularly for the UWM and OWC dyads, with poor households at a greater disadvantage than the rich. Of note, the current situation of maternal undernutrition in Bangladesh is similar to that observed in other LMIC^(36,37)^. Various studies found that the wealth index plays a vital role in shaping women’s nutritional status and that mothers from poor households in Bangladesh were more prone to being underweight^(33)^.Social expectations regarding body size, beliefs and cultural practices about food, nutrition, and physical activity may explain the association between overweight status and higher wealth quintiles^(2,17)^. For instance, a recommendation to follow a reduced-fat diet at the household level can reduce the BMI for those with overweight and obesity, but this intervention could increase the risk for underweight members in the same household. In such a situation, prevention programmes should provide health information that promotes the optimal weight for all individuals in the household. For example, an intervention of reduced energy consumption should be implemented for overweight individuals, particularly for urban residents and those belonging to the wealthiest strata^(38)^. In such interventions, the target population needs to be motivated to consume food with fewer calories and to increase physical activity such as walking. Awareness programmes about the consequences of being overweight or obese, including prevention activities, should be available in schools, the workplace and the community. In contrast, a poor socio-economic condition is associated with underweight women in Bangladesh, because individuals in such conditions cannot afford expensive items such as milk, meat, poultry, fruits and other nutritious foods. For these individuals, the focus should be on healthy diets (e.g. consumption of fruits and vegetables) that lead to optimal BMI and other health outcomes for vulnerable households.

We observed that the age and educational status of the mother, the size of the household and administrative division were significantly associated with the prevalence of DBM among mother–child pairs at the household level in Bangladesh. We found that older mothers had an increased risk of DBM compared to younger mothers. This result is consistent with several studies that suggested that the prevalence of overweight/obesity was higher in older age groups than in younger age groups^(1,5)^. Explanation for this includes reduced activity of the mothers as they age, taking in more calories than they require, and slowing of metabolic processes as they age. It was observed that women aged 30 years or older were more likely to be overweight or obese than younger women in Bangladesh^(7)^. Due to sedentary lifestyles and a reduction in metabolic rates, obesity tends to increase with age among women^(7,10)^. A previous study observed that the prevalence of underweight and overweight women aged 15–49 years in Bangladesh was 22·4 % and 14·1 %, respectively. These conditions are crucial for determining the overall health condition of a child, as maternal health status plays a significant role in child health^(39)^. Our study also demonstrated that maternal education was a significant factor for controlling the DBM. Various other studies also observed a negative association between higher education and malnourished children, as improved knowledge of healthy behaviours can help parents nurture their children^(40,41)^. This is likely because more highly educated mothers tend to have better knowledge of child health and nutrition and can thus choose healthy foods for their household^(42)^. A study conducted in Bangladesh suggested that secondary or higher education of mothers may have contributed to reducing the risk of DBM in the households studied^(43)^. Another study indicated that discordant mother–child pairs were significantly less likely to occur in households in which the mother had a secondary or higher education than in those in which the mother had no formal education ^(34)^. Furthermore, knowledge of infant and young child feeding practices was also poor among uneducated mothers in Bangladesh, which emphasises the importance of maternal education for better child health, which could contribute to reducing the household-level DBM in Bangladesh^(44)^. Various study showed that underweight is more common among less educated mothers, while the overweight is more concentrated among educated woman. This may be because higher-educated individuals often prefer desk jobs where the occupational sitting time is relatively high, which might contribute to overweight status^(45,46)^. Therefore, target-based educational awareness programmes such as the importance of a balanced diet and sufficient nutrition should be introduced at various levels of society.

This study indicated that the size of the household and the administrative division have important implications for the DBM in Bangladesh. We found that small households were often prone to DBM, probably as due to the lack of extra members in their households, they were often unable to prepare home-cooked meals and tended to use more convenient options (processed foods) that could lead to increased weight^(47)^ Moreover, every member of a small household always tries to feed an excessive amount of food (both home-cooked and processed foods) to the youngest member (children) to show their love and affection in the Bangladeshi context, which puts them at an even higher risk of being malnourished^(48)^. Although this study did not attempt to explain these findings, increasing maternal and child overweight/obesity may be an important factor. Therefore, further investigation should be conducted. Regarding administrative divisions, we found that households located in the Khulna and Rangpur divisions were less likely to develop the DBM. Khulna and Rangpur are considered high-performing in various health indicators, such as literacy rates, high maternal nutrition, low mortality rate, low fertility rate, low childhood malnutrition and high socio-economic status^(40)^. In terms of wealth index, we observed that richer and richest households were more likely to generate DBM characterised by OWM & SC/WC/UWC. Our results are similar to many previous studies which have documented a significant positive relationship between the wealth index and household-level DBM^(17,18)^. It was observed that UWC are more prevalent among poorer households, while being overweight is more common among wealthy mothers in Bangladesh, which is also in line with other settings ^(45,46)^. It may be due to having access to Western or fast food, higher occupational sitting time, and excess energy intake, which often lead to overweight and obesity among mothers ^(49,50)^.Various studies also indicated that children from disadvantaged households in Bangladesh are often prone to being stunted, wasted and underweight, while OWC are more concentrated among the wealthiest households^(1,9,40)^.Therefore, policy should focus the mitigation of the unequal wealth distribution for tackling the DBM issues from all strata of society. Our findings and those of other studies suggest that it is high time for policymakers and public health professionals to take the necessary steps to prevent and control the DBM among Bangladeshi women. However, it is quite challenging to implement an intervention in a country in which both undernutrition and overnutrition coexist, as an intervention to address one problem might exacerbate the other. Therefore, target-specific interventions must be formulated and implemented, and health literacy should be encouraged so that people can make the best decisions for themselves given their individual circumstances. The government should sponsor initiatives to educate and encourage affluent women: to embrace a healthy lifestyle and generate awareness of the health impact of being underweight or overweight using mass media; to refashion transport facilities, particularly in urban areas, by making footpaths; and to provide a safe environment for women and adolescent girls to perform physical activities. Physicians and community health workers also can advise their patients, especially pregnant women, so that women receive counselling about weight management before or during early pregnancy. The government should also take the initiative to restrict the production, purchase, and advertisement of junk food, as well as make fruits and vegetables accessible and affordable to people from all socio-economic groups. Furthermore, the development of comprehensive surveillance systems at the household, regional and national levels should be prioritised to tackle the DBM in Bangladesh.

### Strengths and limitations

This study has several limitations. First, the study was based on cross-sectional data, and so we were unable to establish a causal relationship. Second, in the absence of income or expenditure data, we used a household asset-based wealth index as a proxy to assess households’ economic status, and another limitation regarding this was to use the same criteria to assess wealth status in both urban and rural households. Third, due to unavailability of data, various potential confounders (such as physical activity, caregiving practices, cultural influences, postpartum-weight resolution, food taboos, and more detailed components of nutritional status, such as body composition or biochemical or metabolic status) that might affect the DBM cannot be included in the analysis. Therefore, further exploration is warranted to ascertain the contribution of these potential determinants to the development of various forms of DBM in Bangladesh. Despite such limitations, a strength of the present study was that the data were extracted from a nationally representative demographic and health survey with a large randomised sample and low percentages of missing information; thus, our findings can be considered representative of the entire country. The findings of this study will offer strong insights to policymakers and will help them set target-specific, focused public health interventions to tackle the DBM, which is in line with the goal of the latest National Food and Nutrition Security Policy in Bangladesh.

## Conclusion

The current study indicates the overall prevalence of DBM was about 21 %, with a significantly higher prevalence in urban areas of Bangladesh. Higher inequalities in the DBM were observed among the pair of UWM with OWC, which indicated that poor households were more vulnerable to the DBM. In contrast, a low level of inequality of DBM was observed for OWM with SC, WC and UWC. Therefore, health policymakers, concerned authorities and various stakeholders should stress the prevalence of DBM issues and provide the necessary action to tackle this public health problem in Bangladesh.
